# Microstructure and Mechanical Performance of 3D-Printed Carbon Fibre—PLA-PHA Composites

**DOI:** 10.3390/polym18060771

**Published:** 2026-03-23

**Authors:** David Bassir, Sofiane Guessasma

**Affiliations:** 1 Smart Structural Health Monitoring and Control Laboratory, DGUT-CNAM, Dongguan University of Technology, D1, Daxue Rd., Songshan Lake, Dongguan 523808, China; 2 ENS Paris-Saclay, University Paris-Saclay, Centre Borelli, UMR CNRS 9010, 91190 Gif-sur-Yvette, France; 3 INRAE, Research Unit BIA, de la Géraudière, F-44316 Nantes, France

**Keywords:** carbon fibre–PLA/PHA composite, fused deposition modelling, mechanical properties, microstructure

## Abstract

This research delves into the impact of varying printing angles in the range (0°, 15°, 30°, 45°) on the thermal and mechanical characteristics of carbon fibre–PLA/PHA composites fabricated via fused filament fabrication (FFF). The microstructural arrangement within the 3D-printed PLA/PHA is unveiled through the application of SEM, X-ray microtomography and optical imaging. Tensile loading conditions are employed to extract meaningful mechanical parameters such as Young’s modulus, tensile strength, elongation at break, and mechanical energy, all of which are associated with the printing angle settings. The results indicate that the filaments exhibit a porosity of approximately 3%, while the porosity of the printed structure ranges from 27% to 38%, depending on the printing angle. Tensile modulus in the range 840 to 890 MPa is found not to be highly sensitive to the printing angle. However, tensile strength reaches 37 MPa for a printing angle of 30°. The variations across conditions are limited to approximately 6% in tensile stiffness and 16% in tensile strength. Finite element simulations based on 3D imaging indicate that an effective modulus of the solid phase between 1.6 and 1.8 GPa provides the closest agreement between experimental measurements and numerical predictions. This study presents novel findings concerning the deformation mechanisms associated with different length scales, from filament composite to filament arrangement, in the carbon fibre–PLA/PHA composite. This study highlights that while printing angle has a moderate influence on mechanical response, the overall structural integrity and interlayer cohesion of carbon fibre–PLA/PHA composites remain robust across a wide range of processing parameters, demonstrating their potential for reliable structural applications in additive manufacturing.

## 1. Introduction

Additive manufacturing (AM) encompasses a wide range of manufacturing methods rooted in digital models, capable of producing intricate three-dimensional technical components [1,2]. One such technique within AM is fused filament fabrication (FFF), which finds application in fabricating diverse polymeric structures [3,4,5,6,7]. FFF operates by extruding a polymer filament of a few millimetres in diameter (commonly 1.75 mm or 2.56 mm) and then heating it beyond its glass transition temperature to create filaments ranging from 100 to 400 µm in diameter [5]. This process constructs the 3D structure layer by layer, facilitated by controlled nozzle movement relative to the printing base. Usually, the base moves in the building direction. This filament-based deposition profoundly influences the geometric and mechanical attributes of printed polymers [8,9]. The notion of material continuity is disrupted due to two primary discontinuities within the plane of construction and the building direction [10,11]. Investigating these discontinuities and their repercussions on surface finish, performance reduction, and the nature and extent of associated flaws is a pivotal avenue of research in AM. A direct result of material discontinuity is the mechanical anisotropy observed in 3D-printed polymers, which is well documented in the existing literature [12,13,14,15]. The stiffness and strength losses can be particularly pronounced in the building direction. To mitigate material discontinuity’s impact, various approaches are explored, such as adjusting nozzle kinematics to introduce filament overlap, altering orientation and raster characteristics [16], or manipulating printing temperature, a significant parameter for diminishing defects like process-induced porosity [8,17]. This work employs the latter strategy, investigating the influence of printing temperature on the thermal and mechanical attributes of a blend consisting of Polylactic Acid (PLA) and Polyhydroxyalkanoate (PHA). Despite its potential for medical applications and regenerative medicine, this PLA-PHA blend has not received substantial attention [18]. This blend holds promise for designing 3D-printed implants due to its relative strength, minimal toxicity, and biodegradability in organic environments through hydrolysis. While limited research has focused on the printability of PLA-PHA and their blends, a review by Lee et al. [1] offers an overview of key techniques for 3D printing PLA, PHA, and their blends for medical purposes. In a more targeted investigation, Ausejo et al. [19] conducted a comparative analysis between pure PLA and a PLA-PHA blend, revealing distinctions in mechanical performance and thermal stability during hydrolytic degradation of dumbbell-shaped specimens. A critical aspect of characterizing the microstructure of additively manufactured materials is the accurate identification of their 3D phase arrangement, including process-induced defects. X-ray microtomography is particularly well suited for this purpose, as it enables non-destructive three-dimensional visualization of internal architecture, porosity distribution, and filament morphology across multiple length scales [20,21]. The volumetric datasets obtained can be directly used for quantitative defect analysis and integrated into image-based finite element models, allowing precise correlation between microstructural features and macroscopic mechanical behaviour [22]. Its versatility allows for non-destructive three-dimensional characterization of internal architecture, porosity distribution, and filament morphology, providing volumetric datasets that can be directly integrated into finite element (FE) models or used to validate numerical predictions of mechanical behaviour [20,23,24]. A major advantage of X-ray microtomography is its ability to capture real microstructural features with micrometric resolution, enabling accurate voxel-based simulations and quantitative defect analysis in additively manufactured composites [25].

This study delves into the printability of the carbon fibre–PLA-PHA blend, examining it from structural, physical, and numerical standpoints. Tensile performance is evaluated using a dual approach involving both experimental and numerical methodologies. The CF–PLA/PHA composite combines the mechanical reinforcement of carbon fibres [26] with the biodegradability of PLA/PHA [27,28,29], making it suitable for a range of applications, particularly in lightweight structural components, automotive interior panels, consumer goods, sports equipment, and semistructural 3D-printed parts where enhanced stiffness, strength, and reduced environmental impact are required. In biomedical contexts, its biodegradability [30] and tuneable stiffness could be leveraged for temporary implants, scaffolds, or prosthetic components where controlled degradation and mechanical support are needed [31]. In structural or lightweight engineering applications [32], the composite could be used for load-bearing components, automotive parts, or consumer products where enhanced stiffness and strength are required while maintaining environmental sustainability.

There is currently limited work addressing the link between the microstructure induced by 3D printing and the mechanical performance of CF–PLA/PHA blends, especially with respect to the in-plane orientation represented by the printing angle. The literature work addresses either other types of carbon-reinforced composites [33] or orthotropic properties of other materials [14]. In addition, the integration of 3D microstructural characterization with finite element modelling is not reported for this particular blend, although some attempts were made for other materials [34]. For instance, several studies have reported that the mechanical performance of FFF-printed PLA-CF parts is strongly influenced by printing parameters such as raster orientation, layer height, and build direction, which control fibre alignment, interlayer bonding, and the formation of internal voids [35,36,37,38]. For example, recent works have shown that printing angle and raster orientation significantly affect tensile and flexural properties, with lower printing angles generally promoting better load transfer due to improved filament alignment along the loading direction [36,39]. Other studies have also highlighted that the addition of short carbon fibres can significantly enhance the stiffness and strength of PLA parts, although it may introduce microstructural heterogeneities such as inter-bead voids and fibre–matrix interfacial defects that influence fracture behaviour [37,40]. Despite these advances, most of the existing literature focuses primarily on PLA-CF systems, while studies investigating hybrid matrices such as PLA/PHA reinforced with carbon fibres remain limited [41,42]. In particular, the combined influence of printing orientation and microstructural features on the mechanical behaviour of CF-PLA/PHA composites produced by FFF has not been extensively explored. Therefore, further investigation of this material system is needed to better understand its process–structure–property relationships and to expand its potential applications in additively manufactured composite structures.

In our study, the link between microstructure and experimental evidence is explored by the use of X-ray microtomography images in finite element computation. The overarching mechanical behaviour is ascertained experimentally through assessments and related to the printing angle. To delve into the microstructural aspect, a combination of X-ray microtomography and finite element modelling is employed. By amalgamating these experimental and numerical techniques, robust conclusions are drawn concerning the impact of defects on the mechanical characteristics of the PLA-PHA blend. Therefore, the present study aims to establish a comprehensive structure–property relationship for carbon fibre-reinforced PLA/PHA composites manufactured by fused filament fabrication, with particular emphasis on the role of in-plane printing angle. To the best of the authors’ knowledge, no prior study has combined 3D microstructural imaging and voxel-based finite element modelling to explain the anisotropic mechanical response of CF–PLA/PHA composites as a function of raster orientation. By combining advanced microstructural characterization techniques, including SEM and synchrotron X-ray microtomography, with experimental tensile testing and image-based finite element modelling, this work seeks to bridge the gap between filament architecture, porosity distribution, and macroscopic mechanical performance. Through this integrated experimental–numerical approach, this study provides deeper insight into anisotropic behaviour, stress localization mechanisms, and defect-driven performance limitations, ultimately contributing to the optimization of sustainable high-performance biocomposites for structural additive manufacturing applications.

## 2. Materials and Methods

The feedstock is a thermoplastic composite filament (ColorFabb, Limburg, NL, USA) composed of 20% carbon fibres in a blend of Polylactic Acid (PLA) and Polyhydroxyalkanoate (PHA). Supplied as 1.75 mm diameter wire, its key properties include a density of 1.35 g/cm^3^, a melting temperature of 170–175 °C, a Young’s modulus of 3 GPa, and a tensile strength of 40 MPa.

Dogbone tensile specimens (80 × 10 × 4 mm^3^) were printed on a MakerBot Replicator 2 using fixed parameters: a 0.4 mm nozzle, 0.2 mm layer height, 100% infill, 40 mm/s speed, and a printing temperature of 210 °C. The critical variable was the printing angle (Θ), which was defined in a former study as the filament orientation within the plane of construction, i.e., in-plane orientation [16]. This quantity was varied from 0° to 45° with a step of 15°. This ensures a quantitative evaluation of the effect of printing orientation on the microstructure and mechanical response. However, increasing the printing angle beyond these values is expected to result in a symmetrical mechanical response with respect to 45°, due to the geometric symmetry of the raster architecture under uniaxial loading. With respect to out-of-plane orientations, printing normal to the specimen length would lead to very poor mechanical performance, as reported in several studies, including the authors’ own previous results [43,44]. Consequently, the present work focuses on printing perpendicular to the specimen thickness in order to maximize the contribution of filament continuity within the plane that includes the loading direction.

Preliminary analysis of the matrix was conducted to obtain the chemical structure and composition using ATR-FTIR spectra of PLA and PHA. The polymer samples were first prepared by cutting small pieces of film or lightly pressing powders onto the clean ATR crystal of a Thermo Scientific FTIR spectrometer. The crystal was cleaned with lint-free tissue and ethanol before each measurement to prevent contamination. OMNIC software (Thermo Fisher Scientific, Waltham, MA, USA, v. 8.2) was used to set the measurement parameters, typically scanning from 4000 to 400 cm^−1^ with a resolution of 4 cm^−1^ and 32 scans for optimal signal-to-noise ratio. A background scan was collected prior to sample measurement. Each sample was placed in full contact with the ATR crystal, and the spectrum was acquired. The resulting spectra could be baseline-corrected and saved in SPA format or exported as CSV for further analysis.

The microstructure was analysed using Scanning Electron Microscopy (SEM), X-ray microtomography [45], and optical microscopy. Sample cross-sections were cut using a sharp blade to avoid deformation. For images out of the plane, no particular preparation was required, nor polishing. No etching or chemical treatment was applied, so the cross-sections represent the as-printed internal structure of the composite. For 3D imaging, no particular preparation protocol was used. Prior to SEM imaging by FEI Verios G4 UC Field Emission SEM equipment (Thermo Fisher), samples were gold-coated to ensure conductivity of the polymeric matrix. Images were captured at a resolution of 1024 × 768 pixels. Quantitative image analysis of SEM micrographs of the filament cross-section was performed to derive both pore and carbon fibre contents. The analysis was performed using image-processing software ImageJ (NIH, Bethesda, MD, USA, v.1.54), where the carbon fibre and pore phases were distinguished from the PLA/PHA matrix based on contrast differences. The surface fraction of the phases was calculated as the ratio between the total projected fibre area and the total phase cross-sectional area.

**Figure 1:**
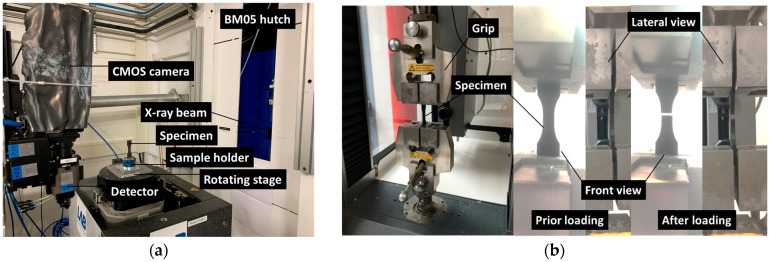
Experimental setup showing (**a**) X-ray microtomography used for 3D microstructure characterization and (**b**) tensile failure progression of a 3D-printed CF-PLA/PHA composite specimen.

Synchrotron X-ray microtomography was also conducted prior to testing for all printing angles (θ = 0°, 15°, 30°, and 45°) at ESRF BM05 beamline (Grenoble, France; Figure 1a). The photon energy was adjusted to 97 keV, and 5000 radiographic images were required to build the tomograms with a voxel size of 3.04 µm.

Quantitative three-dimensional image analysis of the internal structure was carried out using X-ray microtomography (µCT) images to determine the pore volume fraction within the printed structures. The same image analysis software was employed throughout all processing stages. Pre-processing steps included 3D rotation, cropping, erosion/dilation, and segmentation procedures to isolate pore regions from the surrounding microstructure. Due to resolution limitations, individual carbon fibres could not be segmented; however, light-grey regions within the PLA/PHA matrix indicated the presence of carbon fibres. Based on differences in X-ray attenuation (grayscale intensity), the pore network was identified and separated from the solid phase. Pore content measurements were performed on the entire specimens using an external flooding technique to distinguish the background from the internal porosity. Following segmentation, the volumetric porosity fraction was determined by voxel counting.

Tensile testing of the 3D-printed structures was performed in accordance with the DIN EN ISO 527-1 standard [46] to determine key mechanical properties (Figure 1b): Young’s modulus (E_Y_), tensile strength (σ_T_), and elongation at break (ε_M_), and mechanical energy ($U_{M})$ (Figure 1b). A uniaxial Zwick Roell testing machine (ZwickRoell, Germany), equipped with a 10 kN load cell, was used for all experiments. Tests were conducted at a constant cross-head speed of 10 mm/min until specimen rupture.

Young’s modulus was calculated from the slope of the linear elastic region, defined within an engineering strain interval of 0.025% to 0.25%. The yield stress was automatically determined within the first 25% of the strain data with a sensitivity offset of 1% from the maximum force. An optical camera recorded the deformation process at 30 frames per second (fps) for all tests.

Fracture surfaces of the broken tensile specimens were analysed using Scanning Electron Microscopy (SEM). Additionally, high-resolution optical microscopy (at resolutions up to 2880 × 2160 pixels and magnifications from ×20 to ×500) was employed as a complementary technique to characterize key microstructural features.

A finite element (FE) model was developed directly from the X-ray microtomography data in order to capture the real microstructural features of the 3D-printed material, including filament morphology and porosity [20]. First, segmented tomographic volumes were converted to structural meshes using a voxel-to-element conversion scheme according to a previous study [47], where only the solid phase was meshed, excluding voids. Owing to the high resolution of the 3D tomographic datasets (typical volumes of 3480 × 1514 × 1400 voxels), image downscaling was applied to make the computations tractable. The resulting finite element models contained approximately 178 million degrees of freedom, which remains sufficient to accurately resolve the spatial distribution of stress and strain associated with key printed features, including layer stacking through the sample thickness and filament crossings within the plane of construction. All computations were performed using ANSYS software (ANSYS Inc., Canonsburg, PA, USA, v. 2016). Volumetric meshing was carried out in ANSYS Mechanical using 3D tetrahedral elements with three degrees of freedom per node, corresponding to the translations in the main special direction. The material behaviour of the solid phase was adapted according to a micromechanical model, ensuring both the carbon fibre content and the matrix properties to be implemented. The solid phase was modelled as a linear elastic material for small-strain simulations, characterized by its Young’s modulus and Poisson’s ratio, with a mixture law that accounts for the Young’s modulus of carbon fibres (Young’s modulus of 230 GPa, Poisson’s ratio of 0.25), and PLA-PHA matrix (Young’s modulus of 1 GPa and Poisson’s ratio of 0.30, according to data from a previous work by the authors [48]). Perfect bonding between filaments was assumed, consistent with the segmented microstructural representation. Boundary conditions were applied to reproduce the tensile loading conditions of the experiments. For uniaxial tensile simulations, a prescribed displacement was imposed on one face normal to the length, while the opposite face was fixed in the loading direction.

The FE problem was solved using the ANSYS Mechanical solver. Each computation took 1320 s on a workstation equipped with an Intel^®^ Xeon^®^ processor, 256 GB of RAM, running Windows, with post-processing focused on evaluating the local stress and strain distributions within the filaments, identifying stress concentrations around pores and inter-filament interfaces. Effective macroscopic properties, such as apparent elastic modulus, were computed from the reaction forces, which also correspond to the volume averaging of the stress and strain fields over the computational domain.

## 3. Results

### 3.1. Structure and Microstructure of 3D-Printed CF-PLA/PHA Composite

**Figure 2:**
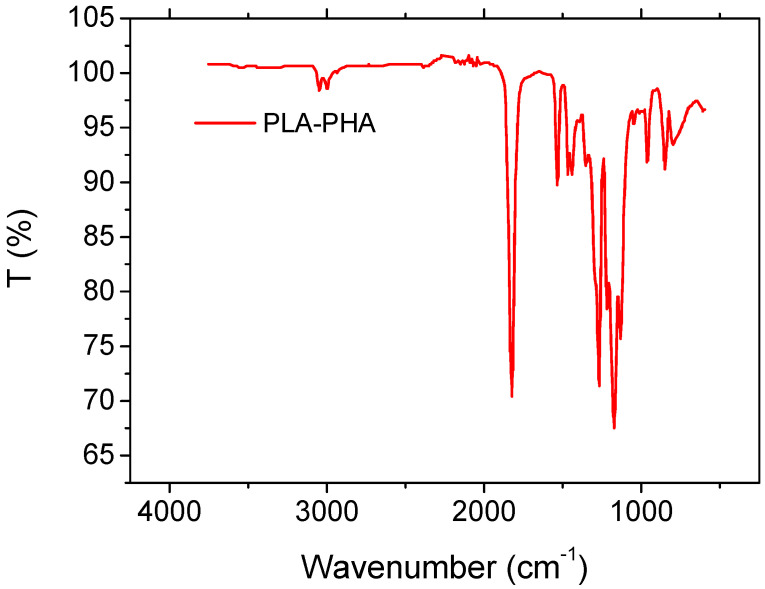
ATR FT-IR spectrum of PLA-PHA material.

The results show that the IR spectrum exhibits the characteristic absorption bands of PLA/PHA-based polymers [49] (Figure 2). The strong peak observed at approximately 1747 cm^−1^ is attributed to the stretching vibration of ester carbonyl groups (C=O), confirming the polyester backbone of the matrix [50]. The absorption around 2995 cm^−1^ corresponds to C–H stretching vibrations of aliphatic –CH_3_ and –CH_2_ groups. Peaks located in the range 1450–1350 cm^−1^ are associated with CH_3_ bending modes, while the bands between 1180 and 1040 cm^−1^ are assigned to C–O–C and C–O stretching vibrations of the ester linkages [51]. Additional peaks observed near 955–870 cm^−1^ are related to amorphous/crystalline phase contributions and backbone vibrations typical of PLA/PHA systems. The FTIR spectrum obtained in this study is consistent with the typical absorption bands reported for PLA [52]. The band observed around 2995 cm^−1^ corresponds well with the reported 2863–3000 cm^−1^ region attributed to C–H stretching vibrations of aliphatic groups. Similarly, the strong peak detected at 1747 cm^−1^ agrees with the characteristic C=O stretching vibration of ester groups in PLA, commonly reported near ~1755 cm^−1^.

The peaks identified in the 1450–1350 cm^−1^ region correspond to C–H bending vibrations, which fall within the typical 1456–1278 cm^−1^ range reported for PLA. Furthermore, the bands observed between 1180 and 1040 cm^−1^ match well with the characteristic C–O and C–O–C stretching vibrations reported near 1182 and 1085 cm^−1^ for PLA.

Finally, the additional peaks detected around 955–870 cm^−1^ are consistent with the bands typically reported near 875 and 758 cm^−1^, which are associated with amorphous and crystalline phase contributions in PLA.

**Figure 3:**
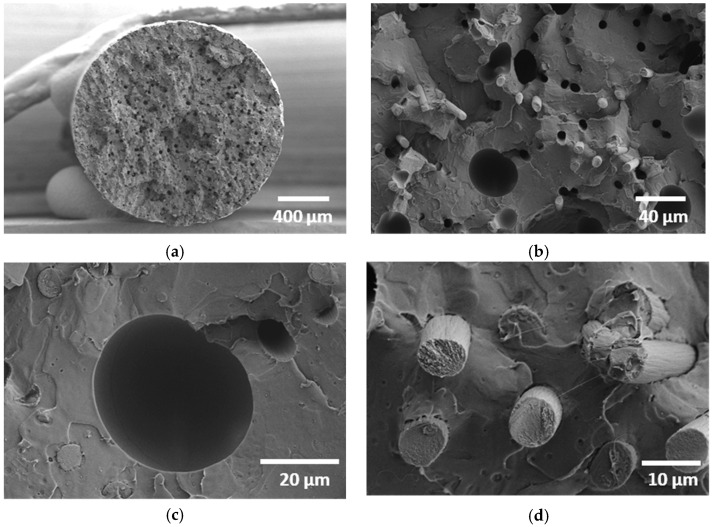
SEM micrographs of the CF–PLA/PHA filament: (**a**) overall view of the filament cross-section highlighting pore populations, including small voids from fibre pull-out and larger matrix pores, (**b**) a zoomed-in view of the spherical pores, (**c**) CF–PLA/PHA interface without significant debonding, and (**d**) fracture surface showing clean fibre breakage with limited debonding.

Figure 3a illustrates the cross-section view of the as-received CF-PLA/PHA composite filament produced via extrusion, where the dark circular or semi-circular domains represent carbon fibre (CF) bundles dispersed within the biodegradable PLA/PHA matrix. The filament cross-section appears as a fairly circular shape with a slight residual roughness. The measured diameter is 1.756 mm. The numerous surface voids of the non-processed filament represent 3.5% of the cross-section area, as determined from 2D image processing. The surface area distribution ranges from 15 µm^2^ to 1200 µm^2^, with an average of 83 µm^2^ and a shape factor of 0.6. A closer examination of the filament (Figure 3b) reveals two distinct populations of pores. The first population is primarily attributed to carbon fibre pull-out following the filament cut. These pores typically measure 70 ± 10 μm, corresponding to the diameter of the carbon fibres. A similar pore morphology was reported by Ning et al. [4] in carbon fibre–reinforced thermoplastic composites fabricated using FFF. Their SEM observations revealed that the extent of porosity increased with increasing carbon fibre loading. The second population is larger (typically 300–500 µm in size) and corresponds to intrinsic voids within the PLA/PHA matrix, which are either formed during filament production or exacerbated by the cutting process. A magnified view (Figure 3c) of the large pores within the filament reveals that they are nearly spherical, with a shape factor of approximately 0.83. Such morphology suggests that these voids are not the result of fibre pull-out or irregular cracking but are instead intrinsic defects within the PLA/PHA matrix. Their smooth, rounded boundaries further indicate that they likely formed as trapped gas bubbles during filament extrusion and were later exposed or enlarged by the cutting process. A closer inspection of the CF–PLA/PHA interface (Figure 3d) reveals the absence of pronounced interfacial gaps, qualitatively supporting the presence of strong fibre–matrix bonding. The carbon fibres appear cleanly fractured rather than pulled out, which is consistent with efficient stress transfer across the interface. The carbon fibres have a nearly circular cross-section with a typical diameter of 7.8 ± 0.5 µm. A preliminary estimation of the volume fraction from SEM micrographs indicates that carbon fibres constitute about 10% of the filament cross-section, which is twice the weight content provided by the supplier. The calculated fibre volume content, derived from the measured composite density using the rule of mixtures and assuming constituent densities of 1.2 g/cm^3^ (PLA/PHA) and 1.8 g/cm^3^ (carbon fibre), is approximately 14%. The 4% reduction in measured fibre content can be directly correlated with the extensive fibre pull-out observed in the SEM micrographs. This indicates that a significant portion of fibres were not bonded within the fracture plane and were instead mechanically extracted during failure, thus not contributing their full reinforcing potential at the critical interface.

**Figure 4:**
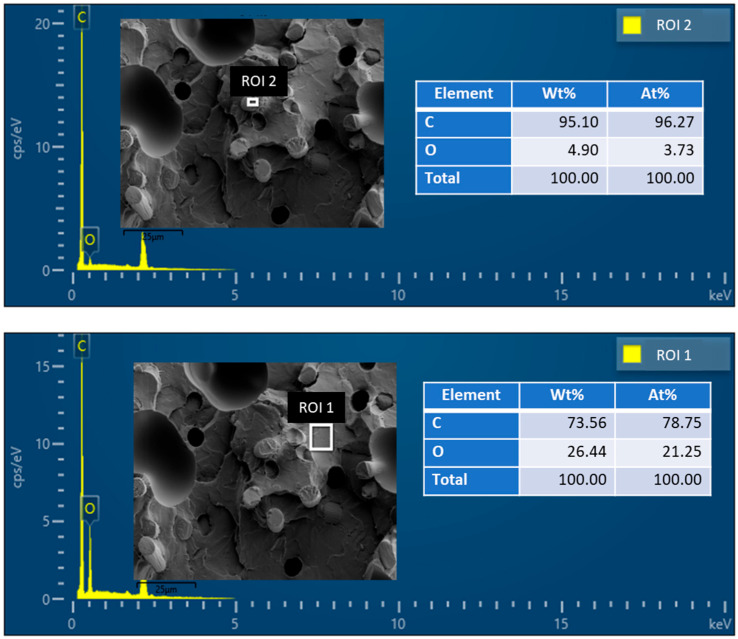
Elemental composition for carbon fibre–PLA/PHA microstructure using EDS.

Figure 4 shows the energy dispersive spectroscopy (EDS) phase map collected with SEM surface imaging of the CF-PLA/PHA composite, illustrating the elemental distribution across the material surface. Quantitative EDS analyses were performed at two regions of interest (ROI 1 and ROI 2) to assess local compositional variations.

In ROI 1, the composition is found to be 73.56 wt% carbon and 26.44 wt% oxygen (78.75 at% C and 21.25 at% O), indicating the presence of the PLA/PHA matrix with characteristic oxygen content. In contrast, ROI 2 shows a composition of 95.10 wt% carbon and 4.90 wt% oxygen (96.27 at% C and 3.73 at% O), consistent with the carbon fibre-rich region. These results confirm the heterogeneous distribution of carbon fibres and the PLA/PHA matrix within the CF-PLA/PHA composite.

**Figure 5:**
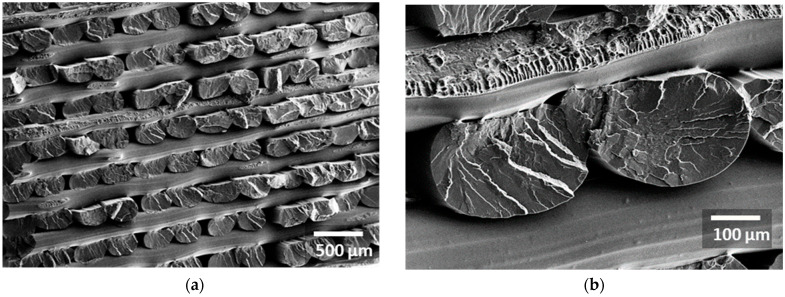

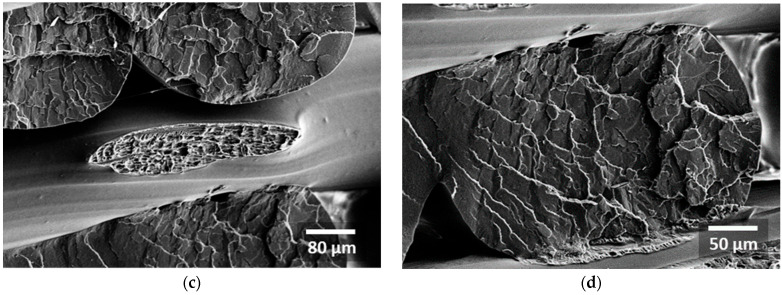
SEM micrographs of the 3D-printed CF-PLA/PHA composite printed at Θ = 15 for different magnifications: (**a**) overview of the filament arrangement along the height, (**b**) higher magnification highlighting necking effect, (**c**) detailed view of filament adjacent pairs, and (**d**) cross-section morphology of the CF-PLA/PHA filament.

Figure 5 shows typical SEM images of the 3D-printed composite at different magnifications. Figure 5a provides a cross-sectional view of the CF-PLA/PHA filament arrangement across the thickness. At the top and bottom of the fractured region, distinct interlayer boundaries are visible, indicating the layered nature of the FFF process. The presence of voids or gaps at these interfaces suggests partial bonding or insufficient diffusion between layers, possibly due to suboptimal temperature or print speed. The observed vertical arrangement is consistent with a 200 µm layer height, featuring filaments within the construction plane oriented in an alternating −30°/+60° pattern. The diameter of these continuous filaments varies from 230 µm down to 160 µm due to compressive forces applied by the deposition nozzle. Additionally, the arrangement of filaments perpendicular to the micrograph’s plane reveals a paired configuration, which generates interstitial voids measuring between 80 and 150 µm between each pair. As shown in Figure 5b, the filament pairs exhibit an elliptical cross-section characterized by a major axis of 300–400 µm and a shape factor of 0.55 ± 0.12. A significant variation in filament height is also observed, with values typically ranging from 150 to 220 µm. Distinguishing the carbon fibre phase from PLA/PHA is challenging due to low contrast and the limited fibre content. Nevertheless, small voids on the filament surface suggest evidence of fibre pull-out. Zooming in on the neck formed by the filament pair (Figure 5c) shows that the laying-down process created a porosity with a distinct triangular shape. This pore has a base length of 190 µm and a typical height of 90 µm. Furthermore, the two filaments are not fully bonded; they exhibit partial cohesion along their height, which accounts for between 55% and 85% of their total minor axis.

On the same micrograph, the debonding area on the axis normal to the micrograph plane shows that the cohesive area between adjacent filaments is elliptical in shape with an aspect ratio as small as 0.22. Figure 5d shows the detailed morphology of the laid-down filament. The cross-section of the CF-PLA/PHA filament can be geometrically simplified as a capsule-shaped profile, consisting of a central rectangle flanked by two semi-circular ends. In this representation, the filament has an overall width of 350 µm and a height of 230 µm, highlighting its flattened form compared to a perfect circle. This simplification is useful because extruded filaments rarely maintain a circular cross-section after deposition; instead, they deform under nozzle pressure and layer compression. By modelling the cross-section in this way, it becomes easier to estimate important geometric properties such as cross-sectional area and perimeter, which are critical for predicting material flow, fibre orientation, and mechanical performance in printed parts.

**Figure 6:**
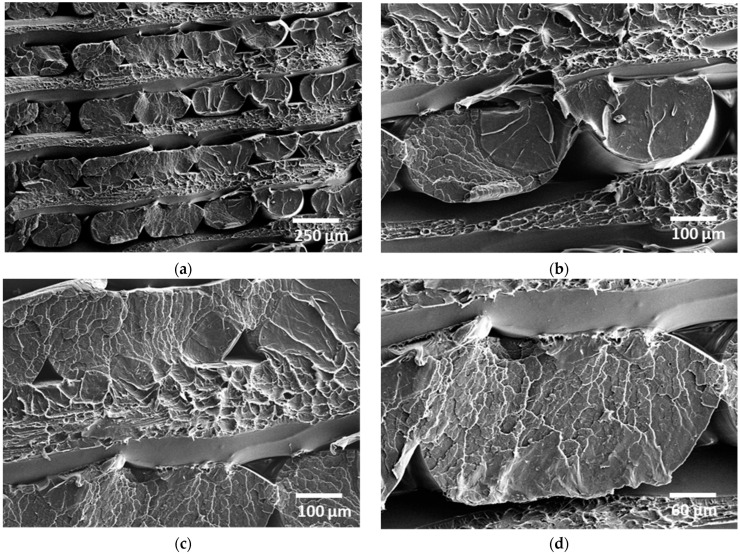
SEM micrographs of the 3D-printed CF-PLA/PHA composite printed at Θ = 30° for different magnifications: (**a**) overview of filament arrangement along the build height, (**b**) higher magnification highlighting the necking effect, (**c**) detailed view of the filament decohesion, and (**d**) cross-sectional morphology of the CF-PLA/PHA filament.

Figure 6 shows the same SEM images of the 3D-printed CF-PLA/PHA composite at different magnifications for a printing angle of 30°. Under this condition, the filament layout exhibits a −15°/+75° filament arrangement. Figure 6a provides a cross-sectional view of the filament arrangement across the thickness, similar to Figure 5a. Although the magnification is slightly higher, the observed vertical arrangement maintains the same 200 µm layer height; however, a larger area of debonding is featured, which results from the higher ratio of filament alignment along the longitudinal direction. Indeed, with the pattern, the filament misorientation with respect to the longitudinal direction is only 15° instead of 30° in the case of Figure 5a. There is no significant variation in the void structure between adjacent filaments. However, a slight increase in the void between layers is noticeable. As shown in Figure 6b, the filament pairs exhibit a fracture pattern, as in Figure 5b, with the slight difference that smooth surfaces appear more likely because of the lower misorientation of the filament with respect to the loading direction. The filament dimension along the largest length is fairly constant and close to 390 ± 15 µm, while the same narrower dimension is 182 ± 23 µm, which makes the aspect ratio close to 0.47. Interstitial voids, on the other hand, measure 93 ± 14 µm at the middle of the layer and extend to 107 ± 41 µm at the edges.

Zooming in on the pulled-out filaments (Figure 6c) shows that the filament connectivity is much higher compared to that in Figure 5c. This is attributed to the small 15°-oriented filament with respect to the direction normal to the loading. The same triangular porosity with a distinct triangular shape is observed, with a typical size of 86 ± 19 µm.

Figure 6d displays a brittle fracture surface characteristic of a zoomed-in filament, with numerous microcracks and river-like patterns spreading across the section. These cracks suggest limited plastic deformation before failure, typical of PLA/PHA under tensile or thermal stress.

**Figure 7:**
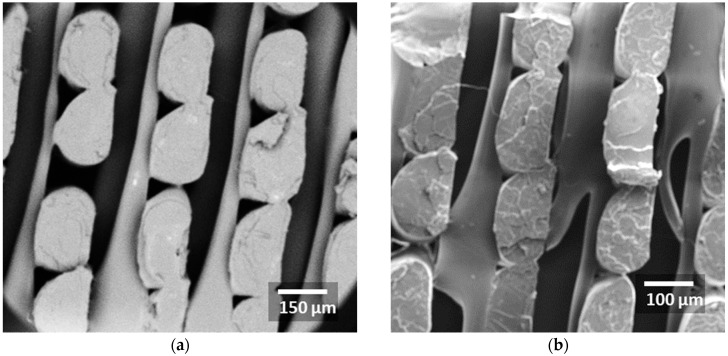
SEM micrographs of the 3D-printed CF-PLA/PHA composite printed at Θ = 45° for different magnifications: (**a**) overview of filament arrangement along the build height; (**b**) higher magnification highlighting the necking effect.

The SEM micrograph in Figure 7a shows the internal structure of the 3D-printed CF-PLA/PHA composite fabricated with a raster orientation of Θ = 45°. The image provides an overview of the filament arrangement along the build height, revealing elongated strands with an elliptical cross-section, aligned normally with respect to the longitudinal direction due to the selected printing angle. A relatively regular spacing between adjacent filaments can be observed, indicating consistent material deposition during printing. The interfaces between neighbouring rasters are visible, and small inter-filament voids are present (Figure 7b), which indicates a relatively low density compared to the other printing angles.

**Figure 8:**
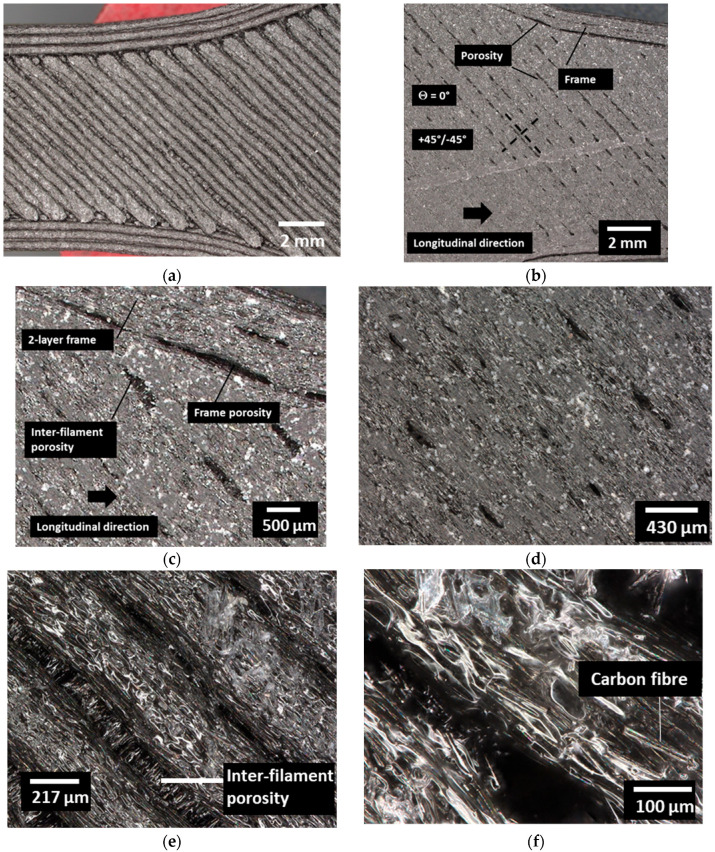
Optical micrographs of the 3D-printed CF–PLA/PHA composite printed at Θ = 0° showing the hierarchical structure of the printed architecture at different magnifications: (**a**) overall macroscopic view of the filament arrangement and raster pattern, (**b**) top view of the printed surface highlighting the alternating ±45° raster orientation and outer frame deposited at 0°, (**c**) magnified view of the frame region illustrating frame-induced porosity and inter-filament gaps, (**d**) detailed view within the raster showing surface discontinuities associated with the necking effect between adjacent filaments, and (**e**,**f**) high-magnification views revealing carbon fibres embedded in the PLA/PHA matrix and their alignment along the filament direction.

Figure 8 exhibits typical optical micrographs detailing the overall structure of the CF-PLA/PHA composite at different magnifications. Figure 8a shows the overall filament arrangement at the macroscopic scale. Both the filaments and the gaps are visible. Slight modifications in the filament paths can also be observed as a result of the change in material flow during printing. Figure 8b presents a zoomed-out view of the top of the 3D-printed CF-PLA/PHA composite printed at 0°. The printing pattern consists of alternating +45° and −45° raster orientations, surrounded by an outer frame deposited at 0°, which enhances structural stability and dimensional accuracy. The longitudinal direction corresponds to the main printing axis, indicating the alignment of deposited filaments along the build path as detailed in the SEM analysis. At this macroscopic scale, small porous regions are visible between filaments, representing inter-bead voids formed due to incomplete fusion or slight variations in extrusion flow and cooling rate. These microstructural characteristics, raster orientation, porosity, and frame geometry collectively influence the anisotropic mechanical behaviour and stress distribution in the FFF part. Figure 8c illustrates a magnified view of the “two-layer frame”. The visualization of this region highlights the “frame porosity”, which is inherent small voids or air gaps that form as a result of the change in the filament path close to the frame. The “inter-filament” porosity adds to the material discontinuities present in the part, which results from the incomplete bonding between the filaments composing the frame. Figure 8d shows a zoomed-in view within the raster, which shows a series of surface discontinuities. These are likely to be the result of the necking effect. Figure 8e,f provide a closer view of the filament arrangement, revealing carbon fibres embedded within the PLA/PHA matrix. The fibres appear well dispersed and predominantly aligned along the main longitudinal direction of the filament, reflecting the shear-induced orientation that occurs during extrusion. This alignment is expected to enhance load transfer efficiency and mechanical performance along the printing direction. In addition, waviness appears to be a common characteristic across all printing conditions. This feature was previously reported by Sanai et al. [53] in 3D-printed CF/Onyx composites. The microstructure also highlights the interfacial bonding between the carbon fibres and the PLA/PHA matrix, which plays a key role in stress distribution and overall composite integrity.

**Figure 9:**
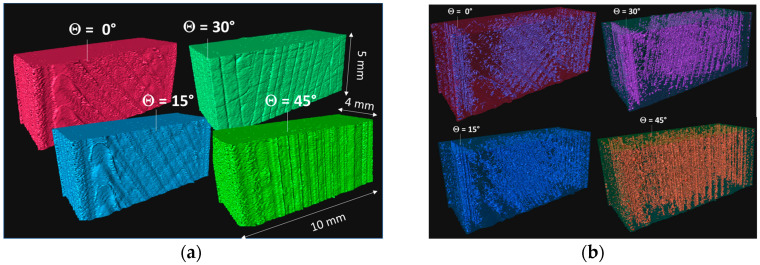
X-ray microtomography results showing (**a**) a 3D rendering of the overall microstructure and (**b**) the porous structure as a function of the printing angle (Θ).

X-ray microtomography results of the 3D-printed CF-PLA/PHA composite are illustrated in Figure 9, which exhibits a 3D rendering of the microstructure as a function of the printing angle. Figure 9a highlights the main features of the printed part in a typical volume of 10 mm × 5 mm × 4 mm, including a two-filament-wide external frame surrounding a core region. Within the core, the filament layups vary with the printing angle θ = 0°, 15°, 30°, and 45°. A systematic 15° shift between printing angles is applied, resulting in layup orientations of −45°/+45°, −30°/+60°, −15°/+75°, and 0°/+90°, respectively. Figure 9b shows the porous network generated by the laying-down process, which exhibits the same patterns as the filament layups. For instance, a porous network is observed within the core of the sample, connected along the filament directions. At the frame, the porous structure aligns along the length of the sample, irrespective of the printing angle. However, a columnar-like porous structure develops in the case of a printing angle of 45°, which corresponds to filaments aligned along the length of the sample. A quantitative analysis of the porosity level as a function of the printing angle is performed, and the results are highlighted in Table 1. The porosity level exhibits a clear dependence on the printing angle θ. As θ increases from 0° to 30°, the porosity fraction rises markedly from 27% at 0° to 38% at 30°. This progressive increase suggests that intermediate printing angles promote less efficient filament packing and reduced inter-road contact, leading to the formation of larger or more numerous interstitial voids within the printed structure. At θ = 45°, however, the porosity drops sharply to a level close to the one at 28%, a value comparable to that observed at 0°. This non-monotonic trend indicates a change in the dominant void-formation mechanism. At higher angles, the filament orientation and layup symmetry likely enhance filament overlap and compaction, improving interlayer bonding and reducing void content despite the inclined deposition paths.

Overall, these results point to an optimal filament arrangement at low (0°) and high (45°) printing angles that limits porosity, whereas intermediate angles (15–30°) are more prone to inefficient packing and higher porosity levels. This behaviour is expected to have a marked impact on the mechanical response of the printed parts, as increased porosity generally correlates with reduced stiffness and strength.

### 3.2. Mechanical Behaviour of the 3D-Printed CF-PLA/PHA Composite

**Figure 10:**
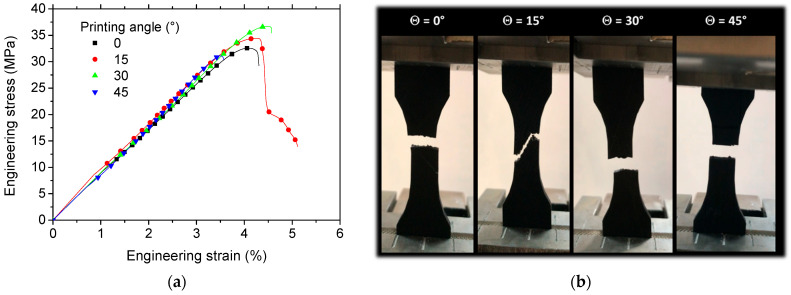
Tensile behaviour of the carbon fibre–PLA/PHA composite wire: (**a**) engineering stress–strain curve and (**b**) corresponding deformation sequence, shown as a function of the printing angle (Θ).

Figure 10 characterizes the tensile behaviour of the 3D-printed carbon fibre-reinforced PLA/PHA specimens, depicting the resulting stress–strain curve alongside sequential snapshots of deformation.

In contrast to the ductile fracture and significant plastic deformation of the pure PLA/PHA matrix, the 3D-printed carbon fibre-reinforced composites fail in a brittle manner, with minimal elongation prior to rupture (Figure 10a). Key quantitative tensile properties are provided in Table 1.

The variability in the measured mechanical properties can be evaluated from the standard deviation values reported in Table 1, which remain relatively low for most parameters, indicating good repeatability of the tensile tests. The porosity fraction (f_p_) shows extremely small standard deviations (±0.01–0.06%), demonstrating that the X-ray microtomography measurements are highly consistent and that the porosity level is well controlled across specimens printed at different angles. For Young’s modulus (E_γ_), the variability is moderate for most printing angles, with standard deviations ranging from ±11 to ±24 MPa for Θ = 0°, 15°, and 30°. This corresponds to a variation of only about 1–3% of the mean value, indicating relatively stable stiffness measurements. However, a larger dispersion is observed at Θ = 45° (±60 MPa), suggesting greater variability in interlayer bonding and filament alignment at this raster orientation, which may lead to less uniform load transfer during tensile loading. The tensile strength (σ_t_) shows very small deviations for most conditions (±0.3–1.6 MPa), representing less than about 5% variation, which indicates good repeatability of the failure stress despite the presence of porosity and microstructural heterogeneities inherent to the FFF process. Similarly, the maximum strain (ε_m_) exhibits slightly higher variability, particularly for Θ = 15° and 45°, likely reflecting differences in local filament bonding and microvoid distribution that influence deformation behaviour prior to failure. Finally, the total mechanical energy (U_m_) presents the largest dispersion among the measured properties (±16–78 mJ/m^3^), which is expected since this parameter integrates both strength and ductility and is therefore more sensitive to local defects such as inter-filament voids or fibre distribution.

The tensile response observed in the present study contrasts with the findings of Ning et al. [4], who reported a more pronounced ductile stage in specimens containing up to 15 wt% carbon fibre in an ABS (Acrylonitrile Butadiene Styrene) matrix.

The tensile properties of the 3D-printed specimens were evaluated as a function of printing angle (Θ) to assess the impact of the manufacturing process. A key finding is that the measured Young’s modulus is approximately three times lower than the supplier’s specification for the raw filament (Table 1). This discrepancy is likely attributable to viscoelastic effects, which were pronounced at the relatively low displacement rate used in this study. When compared with results reported in the literature [54,55], the tensile strength and stiffness obtained in the present study indicate comparatively lower load transfer efficiency than that observed in carbon fibre–reinforced ABS systems.

The fracture behaviour and mechanical performance were highly dependent on the printing angle. As shown in Figure 10b, specimens printed at 0° and 45° exhibited brittle failure with limited deformation prior to rupture. This suggests restricted crack deviation, resulting in a fracture pattern characterized by a pronounced opening mode (transverse cracking perpendicular to the loading direction). In contrast, specimens printed at intermediate angles (15° and 30°) demonstrated greater ductility, leading to increased deformation at break and higher tensile strength. The rupture pattern for these angles more closely followed the raster orientation, with evidence of diffuse damage prior to failure.

The mechanical energy density data across printing angles (Table 1) reveals a compelling and non-monotonic anisotropy profile that provides critical insights into the structure–property relationship of the 3D-printed material. The optimal energy absorption occurs not at the traditional 0° (filament-aligned) orientation, but rather at a 15° offset, where the energy density peaks at 1252 ± 62 mJ/m^3^, a significant 21% increase over the 0° baseline of 1035 ± 16 mJ/m^3^. This peak suggests that a synergistic toughening mechanism is activated at this intermediate angle, likely involving a favourable mix of load transfer: the applied tensile stress is sufficiently resolved into both axial (stretching the filament) and shear (engaging the interlayer matrix) components, as suggested in Figure 10, which promotes distributed deformation and energy dissipation through mechanisms like limited filament pull-out and matrix shear yielding, rather than the brittle, interface-dominated splitting observed at 0°. As the angle increases further to 30° and 45°, the energy density declines, indicating the diminishing benefits of shearing and the growing dominance of the weaker interlayer bond. The accompanying error metrics further inform process reliability, with the low variability at 0° (±1.5%) indicating high manufacturing consistency for aligned prints, while the larger errors at intermediate angles (±5–7%) reflect a more sensitive and variable fracture process.

While the elongation at break for the printed parts is within the lower bound of the raw material’s range, the average tensile strength was approximately 15% lower. The influence of the printing angle on stiffness was not clearly defined, although the 0° angle appeared to produce the lowest values.

**Figure 11:**
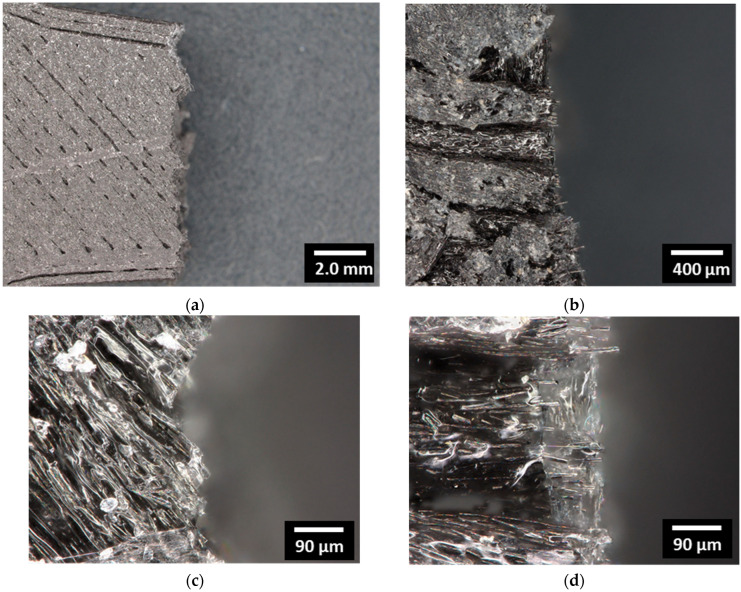
Microstructural analysis of the fracture surface of the 3D-printed CF–PLA/PHA composite printed at Θ = 0°: (**a**) top-view optical micrograph showing the overall fracture pattern characterized by a predominant opening mode with limited crack deviation, (**b**) cross-sectional SEM micrograph highlighting carbon fibre pull-out along the loading direction, (**c**) detailed view showing misoriented filaments within the PLA/PHA matrix, and (**d**) magnified view illustrating fractured carbon fibres embedded in the polymer matrix, indicating effective fibre–matrix adhesion and load transfer during tensile loading.

Figure 11 reveals optical micrographs representing top views of the fractured patterns for the 3D-printed CF-PLA/PHA composite printed at a 0° angle. Limited jaggedness confirms the predominant opening mode, with no signs of multiple cracking along the fractured surface. Figure 11b further reveals several carbon fibres pulled out from the PLA/PHA matrix along the loading direction, indicating fibre–matrix interaction and partial load transfer despite the presence of misoriented filaments, as shown in Figure 11c. The zoomed-in view highlights the extent of carbon fibre contribution to the overall load-bearing capability, as no significant signs of interfacial decohesion are observed (Figure 11d). Instead, the carbon fibres appear fractured, suggesting that the applied stress exceeded the fibre strength rather than causing fibre–matrix separation. This observation confirms an effective interfacial adhesion between the PLA/PHA matrix and carbon fibres, contributing to enhanced mechanical performance and improved energy dissipation during deformation.

The overall porosity content was minimal and exhibited little fluctuation across the range of printing angles. The pores were systematically distributed, aligning with the filament orientation within the raster and the external frame (Figure 5b). This created a consistent porosity profile along the longitudinal direction, which evolved between peak and valley values. Further insight into the microstructure, including the fracture surfaces, is provided by the optical and SEM micrographs in Figure 6.

**Figure 12:**
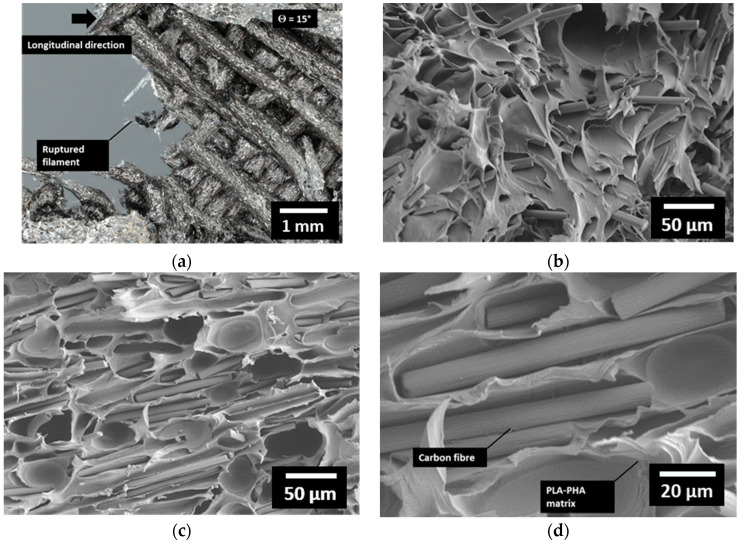
Microstructural analysis of the fracture surface of the 3D-printed CF–PLA/PHA composite printed at Θ = 15°: (**a**) top-view optical micrograph showing a slightly jagged fracture path indicative of mixed-mode deformation, (**b**) SEM cross-section revealing variations in carbon fibre orientation and local fibre–matrix decohesion, (**c**) magnified view of the fibre–matrix interface highlighting partial fibre pull-out and matrix deformation, and (**d**) detailed micrograph showing the formation of interfacial gaps and microvoids in the PLA/PHA matrix surrounding carbon fibres during tensile loading.

For intermediate printing angles such as 15° (Figure 12), a similar observation of a predominant opening mode is still visible at a macroscopic scale. However, the slight jaggedness observed at higher magnification reveals more complex deformation mechanisms, as shown in Figure 12a. An abrupt change in the crack path can be observed, indicating that filament rupture cannot be attributed solely to a combination of shear and uniaxial tension, but rather to localized tearing and heterogeneous stress distribution within the material. A closer examination of the SEM micrographs (Figure 12b) further highlights variations in carbon fibre orientation and the presence of interfacial decohesion between the fibres and the PLA/PHA matrix. This attests to the progressive damage mechanisms occurring during loading, where matrix cracking, fibre pull-out, and interfacial debonding collectively contribute to energy dissipation and the overall failure process of the composite structure. A more detailed view within the deformed composite reveals the extent of matrix deformation occurring in the vicinity of the carbon fibres. Figure 12c,d show the formation of large interfacial gaps between the PLA/PHA matrix and the carbon fibres, which appear partially debonded or pulled out from the surrounding polymer. This separation suggests that interfacial stresses exceeded the adhesion strength, leading to localized decohesion under load. The surrounding matrix exhibits clear signs of plastic deformation and microvoid formation, indicating that stress transfer from the fibres to the matrix was partially maintained before failure.

**Figure 13:**
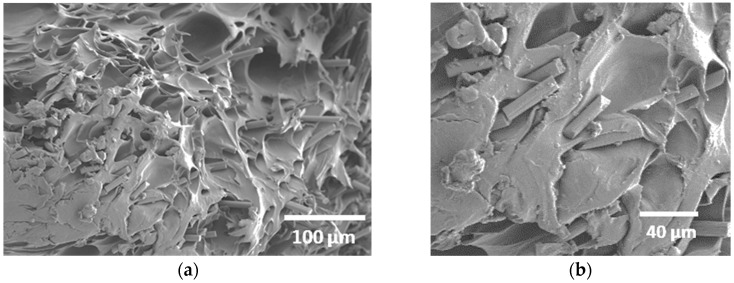
Microstructural analysis of the 3D-printed carbon fibre–PLA/PHA composite printed at 30°: (**a**) zoomed-out view showing carbon fibre pull-out; (**b**) magnified view showing carbon fibre breakage.

A key feature of the fractured surfaces of samples printed at a larger raster angle (30°) is presented in Figure 13a. In addition to the interfacial decohesion observed in other samples, several fractured carbon fibres can clearly be seen (Figure 13b), indicating that the applied stress was efficiently transferred from the PLA/PHA matrix to the reinforcing fibres. This condition corresponds to the highest tensile strength among all tested printing orientations, suggesting that the fibre alignment and interfacial bonding achieved at this angle provided an optimal balance between load transfer and energy absorption, resulting in improved overall mechanical performance.

### 3.3. Link Between the Microstructure and Mechanical Performance of the 3D-Printed CF-PLA/PHA Composite

SEM analysis reveals that the as-received filament possesses an inherent, slightly porous structure. As illustrated in Figure 9, this pre-existing porosity carries over into the printed part and contributes to its reduced mechanical performance. This influence, being an intrinsic material property of the feedstock, is independent of the printing angle. Its effect manifests as a consistent, linear reduction in the tensile properties of the printed material across all tested orientations. Su et al. [56] developed a carbon fibre-reinforced polyamide composite filament with fibre loadings of up to 40 wt.%. Using SEM, they demonstrated that at this high concentration, the composite filament maintained key toughening mechanisms, notably fibre pull-out.

The ranking of the tensile performance of the studied PLA/PHA—CF shows that the average Young’s modulus is of the order of 0.9 GPa, while the tensile strength is 35 MPa. Compared to the authors’ previous PLA baseline [48], the composite exhibits a moderate performance trade-off: stiffness increased by nearly 14%, but tensile strength decreased by 32%. This reduction in strength can be attributed to stress localization phenomena, where defects within the filament or the 3D-printed part act as stress concentrators. This aligns with the findings of Su et al. [56], who reported that increasing carbon fibre content beyond an optimal point can degrade mechanical properties. Their study, for instance, shows that above 30 wt.%, both tensile strength and stiffness decrease—by 8% and 36%, respectively. The authors attribute this reduction to the negative role of porosity and the weak bonding that reduces the load transfer in the composite.

**Figure 14:**
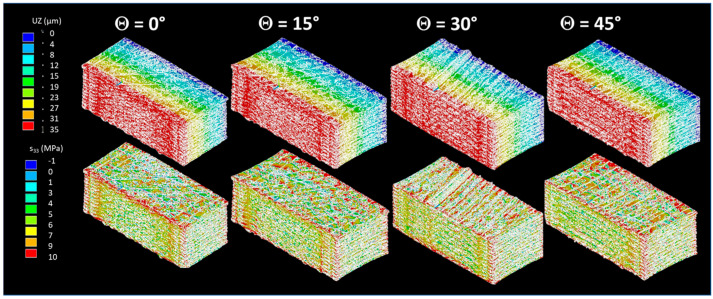
Finite element computations showing the displacement (UZ) and stress (s_zz_) component counterplots as a function of the printing angle (Θ).

On the one hand, the experimental results indicate that the overall stiffness of the 3D-printed composites remains largely unaffected by variations in printing angle, despite differences in filament load transfer relative to the loading direction. On the other hand, microstructural observations reveal that changes in the layup are associated with expected variations in porosity content. To reconcile these two findings, finite element simulations based on X-ray microtomography data are further investigated. Figure 14 shows the nodal solutions detailing the displacement UZ and stress component s_zz_ counterplots for a loading in the z direction as a function of the printing angle. The nodal solutions indicate that the displacement gradient is influenced by the filament architecture through the variability in the printing angle; however, the main characteristics of uniaxial loading are preserved.

A comparison of the axial stress component, s_zz_ obtained from counterplots reveals that variations in printing angle primarily affect the local stress heterogeneity rather than the global stress level. While all configurations exhibit similar average s_zz_ values under identical loading conditions, differences in filament orientation lead to distinct stress concentration patterns, particularly at inter-filament interfaces and around pores. For an applied strain of approximately 0.5% in the Z-direction, most stress values are close to 6 MPa. This corresponds to an effective Young’s modulus on the order of 980 MPa, in good agreement with the experimental results reported in Table 1. Higher local stress levels, reaching up to about 10 MPa, are also observed; however, these remain well below the average tensile strength of 34 MPa, regardless of the printing angle. Specimens printed with filaments aligned closer to the loading direction show a more continuous load transfer and smoother s_zz_ fields, whereas larger printing angles result in increased stress localization and disrupted stress paths. These results indicate that, despite comparable macroscopic stiffness, the printing angle governs the microscale stress redistribution within the material. The identification of the intrinsic properties of the composite filament required to reproduce the experimental results reported in Table 1 indicates that the effective Young’s modulus of the filament ranges between 1.61 and 1.83 GPa. In comparison with the theoretical modulus of carbon fibre–PLA/PHA composites, which can reach up to 35 GPa, this highlights a significant potential for mechanical improvement. Such improvements would require a reduction in porosity within the filament, enhanced alignment of the fibres along the filament axis, improved interfacial bonding between the carbon fibres and the polymer matrix at the filament scale, and a decrease in porosity within the printed raster.

## 4. Discussion

The findings regarding the microstructure of the 3D-printed carbon fibre–PLA/PHA composites and their mechanical behaviour are strongly interconnected. In particular, two key microstructural features—porosity and filament arrangement relative to the loading direction—directly govern the observed mechanical response. The microstructural analysis reveals the presence of two main defect populations, intrinsic porosity within the filament and process-induced inter-filament or interlayer voids, whose distribution depends on the printing angle. This observation is consistent with previous studies. For instance, Ashebir et al. [57] reported that material-extrusion 3D-printed composites exhibit two distinct porosity populations, namely, intra-bead voids caused by incomplete impregnation or gas entrapment and inter-bead voids formed between adjacent deposited filaments. Similarly, Wang et al. [58] demonstrated through X-ray micro-CT and micromechanical modelling that process-induced voids in fused filament fabrication reduce mechanical performance, showing a linear decrease in Young’s modulus with increasing porosity up to approximately 8%. Liao et al. [59] also investigated FFF-printed PLA with processing-induced porosity levels reaching about 32%, comparable to those observed in the present study, and reported a pronounced linear reduction in elastic modulus at porosity levels exceeding 30%. However, analysis of Table 1 indicates that, in the present case, the relationship between porosity and mechanical properties is non-monotonic. The mechanical performance does not decrease linearly with increasing porosity, suggesting that filament architecture and stress transfer mechanisms play a more dominant role than porosity fraction alone in governing the overall behaviour of the composite.

Another pattern identified in this study is the role of intermediate printing angles (15–30°) in driving mixed-mode loading and activating additional energy dissipation mechanisms such as fibre pull-out, matrix shear deformation, and crack deflection. Conversely, at 0° and 45°, the more continuous filament alignment and reduced porosity lead to more direct stress transfer but favour brittle opening-mode fracture with limited crack deviation. These results are not necessarily in line with previous findings regarding 3D-printed monomateials. For instance, Ramírez-Prieto et al. [60] demonstrated that the mechanical behaviour of 3D-printed PLA specimens strongly depends on the raster orientation: specimens printed with a parallel (0°/0°) raster exhibited the highest tensile and flexural strength, while grid (0°/90°) and crisscross (±45°) patterns resulted in lower strength but higher ductility, highlighting the pronounced influence of in-plane printing angle on tensile and flexural performance. Ayatollahi et al. [61] reported that in-plane raster orientation significantly affects tensile and fracture behaviour of FFF PLA parts, with certain angles (e.g., ±45°) yielding improved fracture resistance and elongation at break compared to the typical 0°/90° grid. Therefore, the non-monotonic evolution of tensile strength and energy absorption mirrors the angle-dependent porosity architecture identified from X-ray microtomography.

Finite element simulations reveal pronounced stress localization, particularly at intermediate printing angles where shear stresses are combined with uniaxial tensile deformation. Such stress heterogeneity cannot be adequately captured at the design stage without explicitly incorporating microstructural features, such as those obtained from X-ray microtomography. For example, Issametova et al. [62] employed a 3D thermomechanical finite element model to predict stress fields arising from printing parameters, demonstrating the presence of tensile and compressive zones correlated with the macroscopic structure of the printed part. Although their approach successfully described the overall stress state, it did not resolve stress heterogeneity at the scale of individual filament arrangements. Similarly, Raja et al. [63] conducted finite element simulations to analyse stress, strain, and deformation distributions in 3D-printed PLA under mechanical loading. While their results highlighted the influence of design and support conditions on stress fields, local variations induced by filament architecture and stress concentrations near defects were not explicitly accounted for.

## 5. Conclusions

The printing angle (Θ) exhibited a limited overall influence on the tensile performance of the fused filament fabricated (FFF) carbon fibre–PLA/PHA specimens.

For instance, stiffness was largely unaffected, with Young’s modulus averaging less than 0.9 GPa across all angles. The lowest elongation at break and tensile strength values were observed at the extreme angles of 0° and 45°. However, even these values represent only a modest reduction compared to the raw filament properties—approximately 19% for elongation and 15% for strength. The optimal ultimate properties were achieved at intermediate printing angles between 15° and 30°.

Fracture patterns showed a weak correlation with the raster orientation, particularly at 0° and 45°. Optical and SEM analyses revealed a low porosity content, which is posited to minimize the typical effect of filament orientation on fracture properties. The dominant failure mechanisms identified were interfacial decohesion between the carbon fibres and the PLA/PHA matrix, accompanied by ductile rupture of the polymer matrix itself, and carbon fibre fracture. Finite element results demonstrated that the printing angle mainly affects local stress distribution rather than average axial stress, with most szz values around 6 MPa for a loading of 0.5% (effective modulus ~980 MPa). Filaments aligned with the loading direction show smoother stress transfer, while larger angles increase stress localization. The filament modulus (1.61–1.83 GPa) is far below the theoretical 35 GPa, highlighting potential improvements via reduced porosity, better carbon fibre alignment, and stronger fibre–matrix bonding.

## Figures and Tables

**Table 1:** Tensile properties of 3D-printed carbon fibre-PLA/PHA specimens as a function of printing angle (Θ) and compared to the porosity level issued from X-ray microtomography.

Θ (°)	f_P_ (%)	E_Y_ (MPa)	σ_T_ (MPa)	ε_M_ (%)	U_*M*_ (mJ/m^3^)
0	27.39 ± 0.03	842 ± 16	33 ± 0.8	3.9 ± 0.05	1035 ± 16
15	33.51 ± 0.05	889 ± 24	35 ± 1.6	4.3 ± 0.30	1252 ± 62
30	37.53 ± 0.06	885 ± 11	37 ± 0.3	4.4 ± 0.17	1128 ± 78
45	27.99 ± 0.01	890 ± 60	32 ± 0.8	3.6 ± 0.38	781 ± 67

## Data Availability

The original contributions presented in this study are included in this article. Further inquiries can be directed to the corresponding author(s).

## Abbreviations

The following abbreviations are used in this manuscript:

ABS	Acrylonitrile butadiene styrene
FFF	Fused filament fabrication
PLA	Polylactic Acid
PHA	Polyhydroxyalcanoate
SEM	Scanning Electron Microscopy
